# Supplementary material for: Screening of Lentinula edodes Strains for High Polysaccharide Production and In Vitro Antioxidant Activities
Source: J Fungi (Basel). 2025 Apr 30;11(5):347. doi: 10.3390/jof11050347 (PMC12113202; doi:10.3390/jof11050347)
Supplement: Supplementary file 1 [file jof-11-00347-s001.zip › jof-3531097-supplementary.pdf]

Table S1 Mycelial biomass, intracellular and extracellular polysaccharide contents of 18 *L.edodes* strains under shaking and static culture conditions

Strain No.	Shaking			Static		
	Biomas（g）	IPS（mg˙g^{-1}）	EPS（mg˙mL^{-1}）	Biomas（g）	IPS（mg˙g^{-1}）	EPS（mg˙mL^{-1}）
Xin 808	0.24±0.09 bc	17.82±0.20 b	0.36±0.01 g	0.34±0.25 bc	11.07±0.09 kl	0.27±0.02 g
XG2	0.12±0.03 cd	9.96±0.58 f	0.24±0.004 j	0.22±0.04 bc	15.42±0.08 i	0.27±0.004 g
XG3	0.26±0.03 bc	14.82±0.44 cd	0.47±0.02 d	0.41±0.23 ab	21.63±0.27 f	0.41±0.01 d
XG4	0.16±0.05 c	10.86±0.44 ef	0.32±0.03 h	0.24±0.11 bc	11.73±0.05 k	0.42±0.004 d
XG5	0.05±0.01 cd	11.10±0.82 ef	0.20±0.01 k	0.32±0.09 bc	18.46±0.1 h	0.22±0.002 h
XG6	0.03±0.03 d	15.66±0.90 c	0.29±0.005 i	0.30±0.08 bc	21.75±0.73 f	0.3±0.005 f
XG8	0.11±0.02 cd	7.61±0.55 g	0.13±0.004 l	0.30±0.14 bc	10.17±0.06 l	0.1±0.003 i
XG9	0.30±0.02 b	15.66±0.53 c	0.43±0.01 f	0.21±0.08 bc	39.21±0.74 b	0.41±0.01 d
XG12	0.04±0.004 cd	16.01±0.22 c	0.33±0.005 h	0.24±0.04 bc	19.16±0.63 h	0.11±0.002 i
XG13	0.20±0.02 bc	12.07±0.36 e	0.41±0.01 f	0.34±0.11 b	32.48±0.57 c	0.40±0.01 d
XG14	0.05±0.01 cd	10.80±1.85 ef	0.25±0.002 j	0.56±0.08 a	27.26±0.6 d	0.23±0.02 h
XG19	0.12±0.04 cd	12.83±0.54 de	0.42±0.02 f	0.43±0.07 ab	19.16±0.11 h	0.26±0.003 g
XG20	0.15±0.03 cd	21.75±1.75 a	0.47±0.005 e	0.44±0.02 ab	74.94±0.46 a	0.22±0.005 h
XG21	0.26±0.05 b	17.97±0.79 b	0.70±0.01 a	0.19±0.09 bc	20.53±1.13 g	0.51±0.01 a
XG22	0.16±0.01 cd	13.74±1.31 d	0.29±0.02 i	0.16±0.06 c	16.19±1.34 i	0.31±0.01 f
XG24	0.55±0.22 a	10.65±0.52 ef	0.59±0.03 b	0.57±0.27 a	13.63±0.15 j	0.33±0.004 e
XG26	0.18±0.01 bc	17.94±0.41 b	0.44±0.005 ef	0.15±0.02 c	21.03±0.68 fg	0.45±0.01 c
XG30	0.14±0.02 cd	13.51±2.18 de	0.53±0.02 c	0.19±0.04 bc	24.81±0.47 e	0.48±0.01 b

All values are presented as the means of three replicates ± standard deviation (SD). Different lowercase letters adjacent to the values indicate statistically

significant differences between individual strains at $P < 0.05$, as determined by ANOVA followed by the least significant difference (LSD) test (n = 3). This notation applies to all subsequent data presented in a similar manner.

Table S2 *In vitro* antioxidant activities of polysaccharide from 18 strains under shake culture conditions

Strain NO.	DPPH IC_{50} (mg·mL^{-1})		·OH-IC_{50} (mg·mL^{-1})		O_2^--IC_{50} (mg·mL^{-1})	
	IPS	EPS	IPS	EPS	IPS	EPS
Xin808	1.02±0.02 c	10.39±1.02 b	0.93±0.03 i	0.20±0.02 h	13.37±1.53 d	1.88±0.02 e
XG2	0.11±0.01 m	0.39±0.01 n	1.25±0.10 e	0.12±0.03 k	14.80±1.17 c	1.58±0.06 g
XG3	20.32±1.31 a	20.31±1.31 a	0.65±0.08 o	0.20±0.03 hi	12.14±1.08 e	0.37±0.03 j
XG4	0.16±0.01 k	1.22±0.09 g	0.90±0.04 j	0.19±0.01 i	3.02±0.12 h	0.34±0.04 j
XG5	0.01±0.00 q	0.20±0.02 p	1.19±0.08 f	0.07±0.00 l	0.94±0.03 l	0.80±0.05 i
XG6	0.04±0.00 o	0.54±0.05 k	7.26±0.86 a	0.22±0.03 g	24.40±2.50 a	0.76±0.10 i
XG8	0.01±0.00 p	0.35±0.07 o	0.99±0.14 h	0.11±0.01 k	1.66±0.07 j	1.64±0.05 g
XG9	0.41±0.01 f	0.92±0.03 h	0.63±0.40 p	0.22±0.02 gh	0.95±0.02 l	3.05±0.48 d
XG12	0.28±0.01 i	0.45±0.04 m	0.58±0.03 q	0.30±0.02 e	0.67±0.04 m	0.93±0.11 h
XG13	0.56±0.06 d	3.58±0.55 c	1.37±0.08 d	0.25±0.02 f	9.30±0.97 g	8.39±0.92 a
XG14	0.12±0.01 l	0.01±0.07 q	0.88±0.02 l	0.16±0.01 j	1.13±0.05 k	1.76±0.15 f
XG19	0.07±0.00 n	1.87±0.09 d	0.89±0.06 k	0.19±0.03 i	0.01±0.00 o	0.09±0.02 k
XG20	0.21±0.00 j	0.35±0.08 o	0.75±0.01 n	0.34±0.04 d	1.04±0.16 kl	0.41±0.09 j
XG21	0.46±0.01 e	1.35±0.77 f	1.63±0.21 b	0.87±0.05 a	1.95±0.29 i	0.85±0.07 i
XG22	0.37±0.02 g	1.48±0.17 e	1.06±0.12 g	0.21±0.01 gh	15.96±1.20 b	7.82±0.89 b
XG24	0.30±0.04 h	0.66±0.13 j	0.36±0.03 r	0.38±0.01 c	0.22±0.03 n	6.08±0.02 c
XG26	0.28±0.03 i	0.52±0.09 l	0.83±0.08 m	0.42±0.03 b	1.14±0.06 k	0.83±0.08 i
XG30	1.43±0.10 b	0.73±0.13 i	1.60±0.20 c	0.39±0.04 c	11.64±1.23 f	0.05±0.04 k

Table S3 *In vitro* antioxidant activities of polysaccharide from 18 strains under static culture conditions

Stain NO.	DPPH IC_{50} (mg·mL^{-1})		·OH-IC_{50} (mg·mL^{-1})		O_2^--IC_{50} (mg·mL^{-1})	
	IPS	EPS	IPS	EPS	IPS	EPS
Xin808	1.11±0.04 d	11.21±1.58 d	0.47±0.03 i	0.25±0.02 hi	0.65±0.06 l	1.37±0.87 i
XG2	0.61±0.03 e	2.81±0.03 g	0.19±0.01 j	0.16±0.01 k	3.78±0.47 e	1.09±0.53 j
XG3	1.24±0.06 c	2.47±0.05 h	0.75±0.02 h	0.26±0.02 h	0.66±0.04 l	17.85±2.52 a
XG4	0.02±0.00 ij	0.01±0.00 p	0.27±0.01 j	0.27±0.01 g	0.18±0.01 o	2.73±0.26 e
XG5	0.21±0.01 h	12.99±0.03 c	0.29±0.01 j	0.19±0.01 j	4.88±0.75 d	0.57±0.02 l
XG6	0.09±0.00 i	0.338±0.01 n	0.45±0.02 i	0.29±0.04 f	1.09±0.04 j	1.91±0.05 g
XG8	1.19±0.07 cd	22.39±0.37 b	1.04±0.06 f	0.32±0.02 e	11.05±1.43 c	1.56±0.38 h
XG9	1.36±0.09 b	0.48±0.05 m	1.17±0.09 e	0.40±0.03 d	0.69±0.06 l	0.18±0.79 n
XG12	0.01±0.00 j	0.20±0.00 o	2.75±0.08 c	0.05±0.01 m	1.98±0.38 f	10.77±0.01 b
XG13	1.16±0.08 cd	23.79±0.06 a	13.67±1.05 b	0.27±0.04 g	22.62±3.64 b	3.79±0.96 c
XG14	0.07±0.00 ij	1.82±0.01 i	0.01±0.00 k	0.29±0.02 fg	0.52±0.04 m	1.11±0.72 j
XG19	0.32±0.05 g	1.04±0.02 j	0.40±0.02 i	0.22±0.01 i	1.36±0.04 i	0.04±0.00 o
XG20	1.56±0.06 a	0.22±0.09 o	0.39±0.03 i	0.10±0.00 l	1.48±0.20 h	0.37±0.07 m
XG21	0.51±0.02 f	5.33±0.02 e	0.99±0.04 f	0.61±0.00 a	0.85±0.01 k	0.71±0.02 k
XG22	0.64±0.04 e	5.25±0.04 e	0.85±0.04 g	0.49±0.02 b	27.25±2.98 a	2.88±0.15 d
XG24	0.09±0.01 ij	3.00±0.01 f	0.26±0.02 j	0.22±0.07 i	0.01±0.00 g	0.01±0.00 o
XG26	1.24±0.04 c	0.84±0.05 k	17.36±2.85 a	0.43±0.02 c	1.77±0.36 n	0.61±0.05 kl
XG30	0.49±0.03 f	0.62±0.11 l	1.51±0.18 d	0.43±0.04 c	0.43±0.38 p	2.39±0.44 f
